# The Structural Basis of *Babesia orientalis* Lactate Dehydrogenase

**DOI:** 10.3389/fcimb.2021.790101

**Published:** 2022-01-05

**Authors:** Long Yu, Qin Liu, Wanxin Luo, Junlong Zhao, Heba F. Alzan, Lan He

**Affiliations:** ^1^ State Key Laboratory of Agricultural Microbiology, College of Veterinary Medicine, Huazhong Agricultural University, Wuhan, China; ^2^ Key Laboratory of Preventive Veterinary Medicine in Hubei Province, Wuhan, China; ^3^ Key Laboratory of Animal Epidemical Disease and Infectious Zoonoses, Ministry of Agriculture, Huazhong Agricultural University, Wuhan, China; ^4^ Department of Veterinary Microbiology and Pathology, College of Veterinary Medicine, Washington State University, Pullman, WA, United States; ^5^ Parasitology and Animal Diseases Department, National Research Center, Giza, Egypt; ^6^ Tick and Tick-Borne Disease Research Unit, National Research Center, Giza, Egypt

**Keywords:** *Babesia orientalis*, lactate dehydrogenase, babesiosis, crystal structure, anaerobic glycolysis

## Abstract

Glycolytic enzymes play a crucial role in the anaerobic glycolysis of apicomplexan parasites for energy generation. Consequently, they are considered as potential targets for new drug development. Previous studies revealed that lactate dehydrogenase (LDH), a glycolytic enzyme, is a potential drug target in different parasites, such as *Plasmodium*, *Toxoplasma*, *Cryptosporidium*, and *Piroplasma*. Herein, in order to investigate the structural basis of LDH in *Babesia* spp., we determined the crystal structure of apo *Babesia orientalis* (Bo) LDH at 2.67-Å resolution in the space group *P*1. A five-peptide insertion appears in the active pocket loop of BoLDH to create a larger catalytic pocket, like other protozoa (except for *Babesia microti* LDH) and unlike its mammalian counterparts, and the absence of this extra insertion inactivates BoLDH. Without ligands, the apo BoLDH takes R-state (relaxed) with the active-site loop open. This feature is obviously different from that of allosteric LDHs in T-state (tense) with the active-site loop open. Compared with allosteric LDHs, the extra salt bridges and hydrogen bonds make the subunit interfaces of BoLDH more stable, and that results in the absence of T-state. Interestingly, BoLDH differs significantly from BmLDH, as it exhibits the ability to adapt quickly to the synthetic co-factor APAD^+^. In addition, the enzymatic activity of BoLDH was inhibited non-competitively by polyphenolic gossypol with a *K*_i_ value of 4.25 μM, indicating that BoLDH is sensitive to the inhibition of gossypol and possibly to its new derivative compounds. The current work provides the structural basis of BoLDH for the first time and suggests further investigation on the LDH structure of other *Babesia* spp. That knowledge would indeed facilitate the screening and designing of new LDH inhibitors to control the intracellular proliferation of *Babesia* spp.

## Introduction

The apicomplexan *Babesisa* parasites parasitize the red blood cells (RBCs) of mammals and birds to cause human and animal babesiosis disease (Brennan et al., 2016). The infectious disease is widespread across the world, frequently associated with enormous economic cost in cattle (Yao et al., 2002; Herc et al., 2018; Otgonsuren et al., 2020). Hosts infected by *Babesia* spp. often show a series of “malaria-like” symptoms, such as fever, anemia, icterus, hemoglobinuria, and even death in severe infection (Liu et al., 1997; de Ramon et al., 2016). Unfortunately, babesiosis lacks commercially available vaccines to prevent the disease, and the current drug for treatment is often associated with high-proportioned recrudescence and adverse outcomes (He et al., 2017; Troskie et al., 2017). Those adverse actions urge the search for novel therapeutic strategies to help against *Babesia* spp. infection.

Lactate dehydrogenase (LDH) is likely derived from malate dehydrogenase (MDH) enzyme, which acts as an essential enzyme related to energy generation in widely distributed microbes, plants, tumor cells, and animal cells (Makler and Hinrichs, 1993; Madern et al., 2004; Boucher et al., 2014; Serganova et al., 2018). The enzyme is serving as a terminase in anaerobic glycolysis pathway, which performs the reversible catalysis from pyruvate to lactate, with nicotinamide adenine dinucleotide (NAD^+^) and its reduced form (NADH) as coenzymes (Dando et al., 2001). Interestingly, many bacterial LDHs catalyze the conversion of pyruvate to lactate (or lactate to pyruvate) by the transition from T-state (tense) to R-state (relaxed), and the balance is disturbed by the binding of substrates or allosteric factors (Iwata et al., 1994; Stieglitz et al., 2004). In contrast, the vertebrate LDHs are usually taken for non-allosteric enzymes, as these enzymes in R-state are the preferential ones (Madern, 2002; Katava et al., 2017).

Apicomplexan parasites rely on anaerobic metabolism to produce ATP for various biochemical processes, and these protozoal LDHs have been considered as ideal potential targets for diagnosis and drug screening (Piper et al., 1999; Dando et al., 2001; Al-Anouti et al., 2004; Cornillot et al., 2012; Witola et al., 2017). In malaria parasites, the *Plasmodium falciparum* LDH (PfLDH) plays a critical role in energy generation of this parasite by controlling the reversible catalysis of pyruvate to lactate using the cofactor NADH or NAD^+^ (Fogg et al., 2008). In *Cryptosporidium* spp., available studies indicate that the apicomplexan parasites *Cryptosporidium parvum* and *Cryptosporidium hominis* lack the traditional tricarboxylic acid (TCA) cycle for aerobic respiration and predominantly rely on anaerobic metabolism to generate ATP for survival. Interestingly, the *C. parvum* LDH (CpLDH) is localized in both the cytosol and the parasitophorous vacuole membrane of the parasite (Zhang et al., 2015). In *Toxoplasma gondii*, two LDHs are differentially expressed in tachyzoite stage and bradyzoite stage, which were identified and named as *T. gondii* LDH1 (TgLDH1) and *T. gondii* LDH2 (TgLDH2) (Yang and Parmley, 1997). In *B. bovis*, BbLDH is expressed both in the cytoplasm of parasites and the membrane surface of infected RBCs and plays a suitable drug target for the development of antibabesial drugs (Bork et al., 2004). In addition, the phenomena of the catalytic reaction of LDHs that was gated by their catalytic pocket loop and of the binding of substrate instead of the co-factor, which results in the closure of the loops, have been revealed in bacteria LDH and LDH-A (Kolappan et al., 2015; Nie et al., 2016).

In the life cycle of *Babesia* spp., asexual multiplication begins with the *Babesia* parasites into the erythrocytes of host. However, the mature erythrocytes lack mitochondria and oxidative enzymes and mainly rely on Embden–Meyerhof pathway to obtain ATP (Zhang et al., 2011). On the other hand, *Babesia* parasites were cultured *in vitro* in a gas mixture of 2% O_2_, 5% CO_2_, and 93% N_2_ and depended mainly on anaerobic glycolysis for ATP supply (Rojas Martinez et al., 2016; Abraham et al., 2018). In *Babesia microti*, similar to *C. parvum*, the parasite also lacks the traditional TCA cycle (no malate dehydrogenase gene), and the glycolytic enzyme BmLDH plays a critical role in ATP supply (Cornillot et al., 2012). Furthermore, these protozoal LDHs could also be responsible for maintaining the intracellular acid–base balance of these parasites. Taken together, the studies indicate that *Babesia* spp. LDHs could serve as a potential drug target for the development of anti-babesial compounds.

Related studies demonstrated that the enzymes from these protozoa were applied as drug targets for oxidoreductase inhibitors, such as gossypol and its structural analogue, pyrazole-based compounds, oxamate, azole-based compounds, and so on (Cameron et al., 2004; Choi et al., 2007a; Vudriko et al., 2014; Rai et al., 2017). These compounds also exhibit strikingly high anti-parasitic activities, especially those compounds with renewed structures (Vivas et al., 2005; Lagana et al., 2019). In *B. bovis*, phenolic aldehyde gossypol dramatically inhibited the catalysis activity of BbLDH with a *K*_i_ value of 0.85 μM, and the value was lower than human LDH-A (HuLDH-A) (1.9 μM) and CpLDH (14.9 μM)), and similar to PfLDH (0.7 μM) and BmLDH (0.67 μM). Interestingly, gossypol treatment also reduced the *in vitro* growth of these parasites with IC_50_ values of 50 μM for *B. bovi*s, 15.3 μM for *P. falciparum*, 11.8 μM for *C. parvum*, and 7.07 μM for *B. microti* (Yu et al., 2001; Bork et al., 2004; Choi et al., 2007b; Zhang et al., 2015; Yu et al., 2019). These reports suggest that LDHs of apicomplexan parasites might act as a future drug candidate for finding new anti-parasitic drugs.

In *Plasmodium*, the crystal structure of *P. vivax* LDH (PvLDH) showed the presence of a five-amino-acid insertion in the active pocket loop that does not exist in other mammalian LDHs, and this feature enables the activity-site cavity of PvLDH to bind the synthetic cofactor APADH (Chaikuad et al., 2005). Interestingly, a similar feature also appears in TgLDH1 and CpLDH (Kavanagh et al., 2004). In *Babesia* spp., the crystal structure of BmLDH has been solved, but BmLDH belongs to a mammalian-like LDH and significantly differs from the protozoan-like BoLDH (Yu et al., 2019). Hence, revealing the structure basis of BoLDH can help in the gap of knowledge about *Babesi*a spp. LDH. In this study, the focus was on *B. orientalis* LDH (BoLDH), and we identified a novel cDNA clone encoding the BoLDH, solved the structure of BoLDH in apo form, and elaborated its structural characteristics and biological functions. Knowing the structural basis of LDH would provide some theoretical guides to screen and design new LDH inhibitors.

## Materials and Methods

### Parasite and Preparation of RNA and cDNA

*Babesia orientalis* (Wuhan strain) positive blood with ~7% parasitized erythrocytes was harvested from the jugular vein of previously experimentally infected water buffaloes and stored in -80°C with the addition of RNA*late*r™ Stabilization Solution (Invitrogen, Carlsbad, CA, USA) in the State Key Laboratory of Agricultural Microbiology, Huazhong Agricultural University, China (Liu et al., 2007; He et al., 2011). The total RNA was extracted from the purified *B. orientalis* merozoites using TransZol Up (TransGen Biotech, Beijing, China) and dissolved with RNase-free DnaseI (TAKARA, Dalian, China). The cDNA was generated from 1 µg of the total RNA by using PrimeScript™ RT reagent Kit with gDNA eraser (TAKARA, Dalian, China) according to the instructions of the manufacturer.

### Preparation of Recombinant Plasmid

Primer pairs for amplifying the full-length BoLDH sequence were designed based on the fragment of BoLDH screened from *B. orientalis* genome database (**Supplementary Table S1**). The PCR reaction was performed at 95°C for 5 min, followed by 95°C for 30 s, 57°C for 30 s, and 72°C for 30 s (35 cycles), and finally at 72°C for 5 min for an extension period. The PCR amplicon was purified by using EasyPure^®^ PCR Purification Kit (TransGEN, Beijing, China) and cloned into a pET-28a expression vector. The two mutant BoLDH plasmids were engineered by using Q5 Site-Directed Mutagenesis kit (NEB, Beijing, China). All the recombinant plasmid sequences were further confirmed by DNA sequencing.

### Protein Expression and Purification

The pET-28a-BoLDH expression vector plasmids and two mutant BoLDH plasmids were separately transformed into *Escherichia coli* BL21 (DE3) competent cells (TransGEN, Beijing, China), and the certified BL21 monoclonal strain was incubated at 37°C in 1 L LB medium containing 100 mg/ml kanamycin (1:1,000) for 3 h. This was followed by induction with 0.8 mM IPTG (Biosharp, Anhui, China) when the culture reached 0.6 to 0.8 density at OD_600_. The cells were cultured for another 12 h at 28°C before harvesting.

For protein purification, the induced BL21 cells were collected by centrifugation at 7,000 rpm for 10 min in a high-speed refrigerated centrifuge (Hitachi, Tokyo, Japan), resuspended in 30 ml His binding buffer (300 mM NaCl, 10 mM Tris-base, 50 mM NaH_2_PO_4_·2H_2_O, 10 mM imidazole, pH 8.0) and lysed by passing through a high-pressure homogenizer at 1,000 bar. After centrifugation at 10,000 rpm for 10 min (4°C), the precipitate was discarded, and the supernatant was collected and filtered through a 0.45-um-pore-size filter. The filtered supernatant was loaded onto Ni sepharose beads (GE Healthcare, Uppsala, Sweden) and eluted with 20–400 mM imidazole (gradient elution). The purified proteins were stored in elution buffer containing 5 mM EDTA and further filtered by using a Superdex 200 gel filtration column (GE Healthcare, Uppsala, Sweden) equilibrated with B2 buffer (20 mM Tris-HCl and 200 mM NaCl, pH 7.4) on AKTA Pure (GE Healthcare, Uppsala, Sweden). For crystallization, the purified rBoLDH protein was concentrated to approximately 13 mg/ml and stored at 4°C.

### Enzyme Kinetics and Inhibition Assays

The rBoLDH activity (pyruvate to lactate or lactate to pyruvate) was measured by monitoring the changes of density of NADH using a microplate reader (Biotek, Vermont, USA) at OD_340_. The forward assays (200 μl total volume) were performed at room temperature (25°C) in 50 mM Tris-HCl buffer (pH 7.5) containing 50 ng rBoLDH, 2,000 μM pyruvate, and 800 μM NADH (Sigma-Aldrich, Shanghai, China). The reverse assays (200-μl total volume) were performed at room temperature (25°C) in sodium carbonate sodium bicarbonate buffer (pH 9.5) containing 50 ng rBoLDH, 200 mM lactate, and 1,200 μM NAD^+^ or APAD^+^ (Sigma-Aldrich, Shanghai, China). The kinetic parameters of rBoLDH were determined by variable substrate and co-factor concentrations (*i*.*e*., pyruvate at 100–3600 μM, NADH at 150–800 μM, lactate at 10–400 mM, and NAD^+^ or APAD^+^ at 100–2,000 μM). The assay for rBmLDH was performed at 25°C in sodium carbonate sodium bicarbonate buffer (pH 9.5) containing 100 ng rBmLDH, 50 mM lactate, and 0.2–2 mM APAD^+^.

Gossypol (Sigma-Aldrich, Shanghai, China) was prepared as 0.2-M stock solution in dimethylsulfoxide (DMSO) and further diluted with double-distilled water. The inhibition effect of gossypol against rBoLDH activity was determined at room temperature in sodium carbonate sodium bicarbonate buffer (pH 9.5) containing 200 mM lactate, 100–2,000 μM NAD^+^, and 5 μM of gossypol. The data were fitted to the Michaelis–Menten equation to determine the *K*_M_ and *V*_max_ values using GraphPad prism5 software (La Jolla, CA, USA), and the catalytic constant, *k*_cat_, values were calculated according to the following equation: *k*_cat_ = *V*_max_/*E*_t_ (*E*_t_ is the concentration of enzyme sites). The final concentrations of DMSO have no effect on the rBoLDH catalysis in a preliminary experiment, and all the experiments were repeated three times.

### Crystallization

For initial screening of crystallization conditions, the crystallization experiment of rBoLDH apo form was performed by the hanging drop method of vapor diffusion, and crystal buffer (572 conditions) from the initial screening kit (Hampton, CA, USA) was used. Briefly, 0.5 μl rBoLDH was mixed with 0.5 µl reservoir solution in 48-well plates. The crystal plates were placed at 20°C constant temperature, and crystal growth was determined by viewing with a microscope every day. The crystal conditions of rBoLDH were further optimized, and the optimal conditions were determined as 1 M lithium chloride, 0.1 M sodium acetate, and 25–30% (w/v) PEG 6000.

### Data Collection and Processing

The crystals of rBoLDH were cryoprotected in liquid nitrogen with the mother liquor and 25% glycerol for data collection. The X-ray datum of rBoLDH was collected at Shanghai Synchrotron Radiation Facility at beamline BL17U (wavelength = 0.97732 Å). The reflections were integrated, merged, and scaled using HKL-3000 software, and the crystallographic data for rBoLDH are shown in **Table 1**. The structure of rBoLDH was solved by the molecular replacement method with the structure of apicomplexan LDH-like MDH (ancestral sequence) as template (PDB accession no. 4plc) (Boucher et al., 2014). Density modification was performed using COOT 0.8.2 EL software (ccp4) and refined using PHENIX version 1.12-2829-000.

**Table 1:** Statistics of data collection and refinement.

	BoLDH (apo)
Data collection	
Space group	*P*1
Cell parameter [*a*, *b*, *c* (Å)]	81.9, 92.3, 109.9
α, β, γ (°)	77.5, 70.7, 64.4
Wavelength	0.97732
Resolution range (Å)	44.997–2.671
Completeness (%)	93.55%
*R*_merge_ (last shell)	0.078 (0.503)
*I*/*σ* (last shell)	17.1 (4.872)
Redundancy (last shell)	3.2 (3.1)
Refinement	
Resolution (Å)	44.997–2.671
*R*_work_/*R*_free_ (%)	23.6/26.8
Number of reflections	72,344
Number of protein atoms	35,845
Number of solvent atoms	–
Average *B*, all atoms (Å^2^)	26
RMSD	
Bond length (Å)	0.002
Bond angle (Å)	0.472
Ramachandran plot (%): favored, allowed outliers	95.83, 413, 0.04

The highest-resolution values are written within parentheses.

R_merge_ = ΣΣ |I_i-_<I>|/Σ ΣI_i_, where is I_i_ the intensity measurement of reflection h and <I> is the average intensity from multiple observations.

R_work_ = Σ||Fo| - |Fc ||/Σ|Fo|, where Fo and Fc are the observed and calculated structure factors, respectively.

R_free_ is equivalent to R_work_ but where 5% of the measured reflections have been excluded from refinement and set aside for cross-validation.

### Sequence Analysis and Structure Comparisons

The BoLDH amino acid sequence was aligned with the available *B. bovis* LDH (BbLDH; XBBk016131) using Multalin version 5.4.1 and further analyzed by CD-search (Conserved Domain Search Service) of NCBI for confirming the presence of LDH catalytic center (https://www.ncbi.nlm.nih.gov/Structure/cdd/wrpsb.cgi). Multiple sequence alignment and phylogenetic analysis based on the amino acid sequence of BoLDH and related apicomplexan and mammalian LDHs were created by using MEGA7 software. The root mean square deviation (RMSD) values were evaluated using the PDBe Fold service on the European Bioinformatics Institute website (http://pdbe.org/fold/).

### Molecular Docking and Simulation

To determine a ligand–receptor binding mode, we used a CDOCKER program to create the binding modes for gossypol. Before running the program, the structure model of BoLDH was optimized by using the Discovery Studio 2018 Client software version 18.1.100.18065, including removing water molecules and dopant atoms, cleaning geometry, adding hydrogen atoms, and defining the active site. High-temperature kinetics was used to find the flexible conformational spaces of ligands, and the method of simulated annealing was used to optimize conformations in the active site of the receptor. The values for top hits, random conformations, and orientations to refine were set as 10, respectively. In addition, the parameter of simulated annealing was adjusted to true, and all the other parameters were left at the default setting. The structure data format (SDF) files of gossypol and oxamate were obtained from PubChem under the accession number 24895349 and 57654482 (https://pubchem.ncbi.nlm.nih.gov), and software permission was provided by the State Key Laboratory of Agricultural Microbiology, Huazhong Agricultural University, China.

## Results

### A Potential Drug Target for Controlling Babesiosis

**Figure 1:**
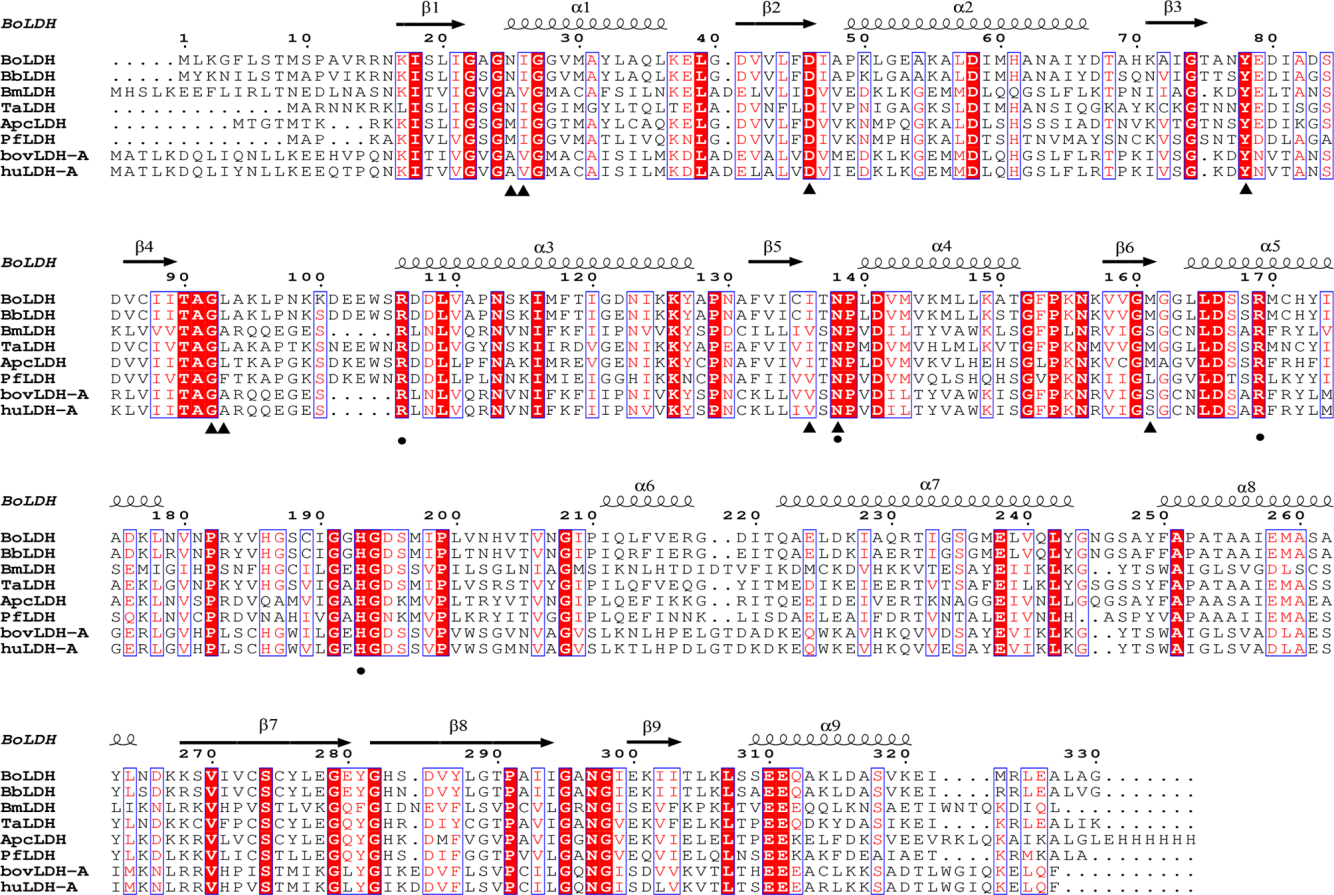
Multiple sequence alignment based on amino acid sequences of BoLDH and related LDHs. BoLDH sequence were aligned with other protozoans and mammals, and the multiple sequence alignment were created by ClustalW. Black triangles indicated the key residues for NADH, and black solid circles indicated the key residues for substrate analog oxamate. The residues were labeled based on the holo PfLDH (PDB accession number 1t2d, Cameron et al., 2004).

The full-length open reading frame (ORF) nucleotide sequence of BoLDH was obtained from *B. orientalis* cDNA by PCR method with a total size of 999 bp and encoded a polypeptide of 330 amino acid residues. The multiple sequence alignment analysis of the BoLDH with the apicomplexan parasites and mammalian LDHs displayed that the amino acid sequence of BoLDH shares a high identity of 65.45, 48.79, and 56.6% with those of LDHs from *Theileria annulata* (ADG45564.1), *Plasmodium falciparum* (ABH03417.1), and ancestral apicomplexan lactate dehydrogenase (4plc) and a low identity of 27.06, 30.59, and 29.41% with *B. microti* (MN102392), *Bos taurus* LDH-A (BAA14171.1), and *Homo sapiens* LDH-A (CAE11711) respectively. However, an amino acid sequence identity of 90% between BoLDH and BbLDH (XP_001611047.1) was exhibited (**Figure 1**). Furthermore, a five-amino-acid-residue insertion (position 101-105, —DEEWS—) was also observed in BoLDH, similar to other protozoa (except for BmLDH) and different from its mammalian counterparts.

### Preparing High-Quality Recombinant BoLDH

**Figure 2:**
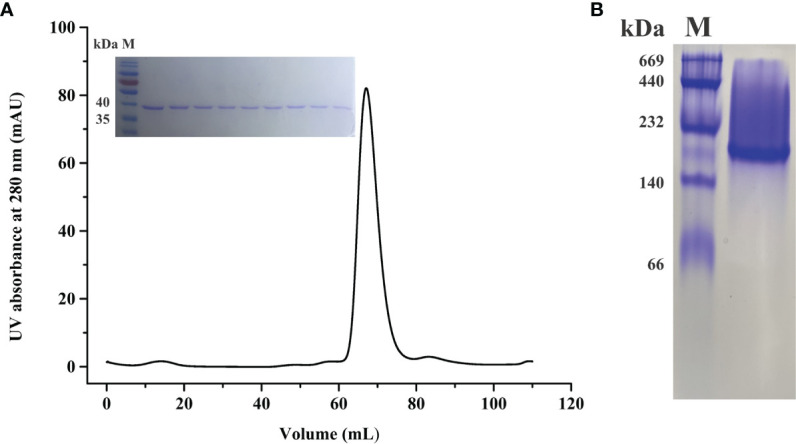
Molecular sieve chromatography and protein gel analysis for rBoLDH. **(A)** A representative peak diagram of sieve chromatography (Superdex 200 pg). The illustration showed the separation effect of rBoLDH examined by 12% SDS-PAGE. **(B)** 8% Blue native-PAGE analysis for rBoLDH. Lane M: High molecular weight marker (GE Healthcare, Uppsala, Sweden).

To explore the structural basis of BoLDH, the full ORF of BoLDH was expressed in *E. coli* with a His6 tag on the N-terminus. The rBoLDH was purified by Ni Sepharose 6 Fast Flow and further filtered using a Superdex 200 gel filtration column. The result of sieve chromatography showed that the rBoLDH was isolated from recombinant protein eluent with an appearance time of ~60 ml (**Figure 2A**). Blue native-PAGE (BN-page) showed that rBoLDH is a tetrameric enzyme in the absence of sodium dodecyl sulfate and has a ~160-kDa size (**Figure 2B**). The His6 tag on the N-terminus was not removed for the subsequent crystallization experiment.

### Enzyme Kinetics of rBoLDH and Inhibition Constant

**Figure 3:**
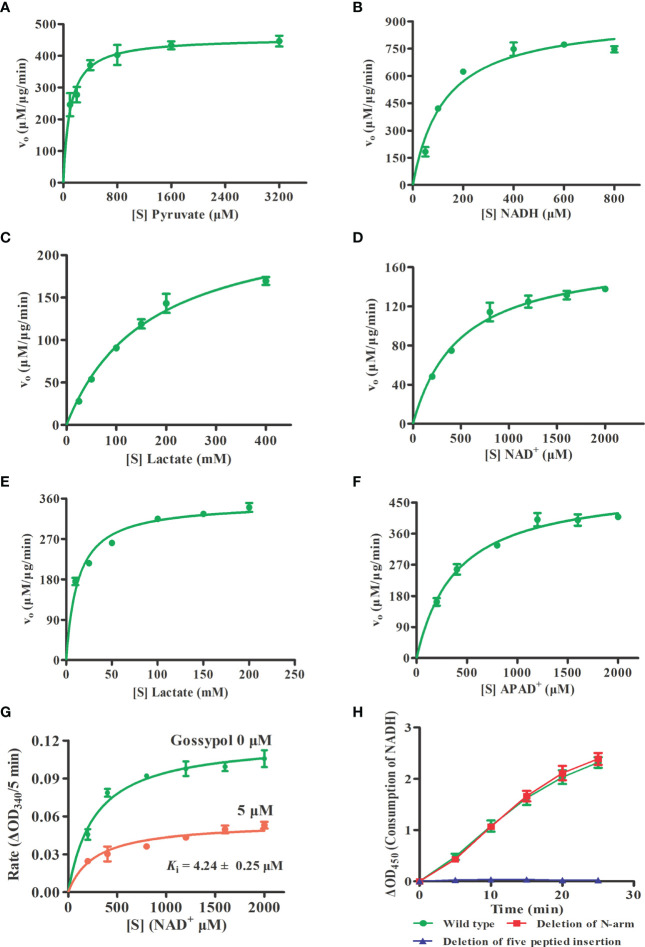
Enzyme kinetics and inhibition constant. **(A, B)** Michaelis-Menten Kinetics for the forward reaction of rBoLDH. Variations of rBoLDH activity with concentration of pyruvate and NADH. **(C, D)** Michaelis-Menten Kinetics for the reverse reaction of rBoLDH. Variations of rBoLDH activity with concentration of lactate and NAD^+^. **(E, F)** Michaelis-Menten Kinetics for the reverse reaction of rBoLDH. Variations of rBoLDH activity with concentration of lactate and APAD^+^. **(G)** Michaelis-Menten-based kinetics of inhibition of rBoLDH activity in the presence of 5 µM gossypol. The changes of density of NAD^+^ were monitored at OD_340_. **(H)** Enzyme activity test for two variants of rBoLDH. The forward assays were performed at 25°C in pH 7.5 50 mM Tris-HCl buffer, containing 50 ng rBoLDH, 2 mM pyruvate and 0.8 mM NADH. S, substrate as indicated; V_o_, initial velocity. The error bar represents mean ± SD (n=3), and Non-linear regressions and XY graph were drawn with GraphPad5.0.

To characterize the enzyme kinetics of BoLDH, the ability to catalyze the reversible conversion of pyruvate to lactate using β-NADH or NAD^+^ was monitored *in vitro*. For forward reaction, the Michaelis–Menten kinetics on pyruvate and β-NADH are shown in **Figures 3A, B**. The Michaelis constant *K*_M_ of BoLDH for pyruvic acid and β-NADH were 102 ± 19.2 and 126 ± 19.9 μM, respectively. At pH 7.5, the catalytic efficiency for pyruvate was found to have a *k*_cat_ value of 56.5 S^-1^, a *V*_max_ value of 458 ± 17 μM μg^-1^ min^-1^ and a *k*_cat_/*K*_M_ value of 5.54 × 10^5^ S^-1^ M^-1^. The rate for β-NADH had a *k*_cat_ value of 115 S^-1^, a *V*_max_ value of 929 ± 42.8 μM μg^-1^ min^-1^ and a *k*_cat_/*K*_M_ value of 9.13 × 10^5^ S^-1^ M^-1^. For reverse reaction, the Michaelis–Menten kinetics on lactate and NAD^+^ are shown in **Figures 3C, D**. The Michaelis constant *K*_M_ of BoLDH for lactate and NAD^+^ were 166 ± 24.2 and 493 ± 69.2 μM, respectively. At pH 9.5, the catalytic efficiency for lactate was found to have a *k*_cat_ value of 30.5 S^-1^, a *V*_max_ value of 247 ± 167 μM μg^-1^ min^-1^, and a *k*_cat_/*K*_M_ value of 1.84 × 10^5^ S^-1^ M^-1^. The rate for NAD^+^ had a *k*_cat_ value of 21.5 S^-1^, a *V*_max_ value of 174.3 ± 8.05 μM μg^-1^ min^-1^, and a *k*_cat_/*K*_M_ value of 4.36 × 10^4^ S^-1^ M^-1^.

Interestingly, we found that BoLDH catalyzes the conversion of lactate to pyruvate using synthetic APAD^+^ instead of NAD^+^ as a cofactor, and the Michaelis constant *K*_M_ for lactate and APAD^+^ was 12.9 ± 1.49 and 395 ± 49.9 μM, respectively. At pH 9.5, the catalytic efficiency for lactate was found to have a *k*_cat_ value of 43.3 S^-1^, a *V*_max_ value of 351 ± 8.27 μM μg^-1^ min^-1^, and a *k*_cat_/*K*_M_ value of 3.36 × 10^6^ S^-1^ M^-1^. The rate for APAD^+^ had a *k*_cat_ value of 62.3 S^-1^, a *V*_max_ value of 502 ± 18.8 μM μg^-1^ min^-1^, and a *k*_cat_/*K*_M_ value of 1.57 × 10^5^ S^-1^ M^-1^ (**Figures 3E, F**). The data indicate that BoLDH adapted quickly to the synthetic co-factor APAD^+^, and the efficiency is higher than NAD^+^. All the kinetic parameters are summarized in **Table 2**. In the measurement, BmLDH was used as a control for the comparison of utilization efficiency on APAD^+^, and we found that the Michaelis constant *K*_M_ for APAD^+^ was 880 ± 120 μM. At pH 9.5, the catalytic efficiency for APAD^+^ was found to have a *k*_cat_ value of 4.2 S^-1^, a *V*_max_ value of 34.1 ± 1.98 μM μg^-1^ min^-1^, and a *k*_cat_/*K*_M_ value of 4.78 × 10^3^ S^-1^ M^-1^ (**Supplementary Figure S1**). The result demonstrated that BmLDH has a very low ability to employ APAD^+^ as a co-factor.

**Table 2:** Kinetic parameters of BoLDH on different substrates and cofactors.

Parameter	Pyruvate	NADH	Lactate	NAD^+^	APAD^+^	Lactate (APAD^+^)
*K*_M_ (μM)	102	126	166	493	395	12.9
*V*_max_ (μM μg^-1^ min^-1^)	458	929	247	174	502	351
*K*_cat_ (S^-1^)	56.5	115	30.5	21.5	62.3	43.3
*K*_cat_/*K*_M_ (S^-1^ M^-1^)	5.54 × 10^5^	9.13 × 10^5^	1.84 × 10^5^	4.36 × 10^4^	1.57 × 10^5^	3.36 × 10^6^

Subsequently, the inhibition constant (*K*_i_) of polyphenolic gossypol against the catalytic activity of BoLDH (lactate to pyruvate) was evaluated. As shown in **Figure 3G**, gossypol served as a noncompetitive inhibitor on BoLDH with respect to NAD^+^, and its *K*_i_ value was 4.24 ± 0.25 μM. The value is higher than those of BmLDH (0.67 μM), BbLDH (0.09 μM), and PfLDH (0.7 μM) but lower than that of CpLDH (14.8 μM) (Gomez et al., 1997; Bork et al., 2004; Zhang et al., 2015; Yu et al., 2019). Therefore, BoLDH is sensitive to the inhibition of gossypol and may also be to the derivatives of gossypol.

### Crystal Structure of BoLDH

**Figure 4:**
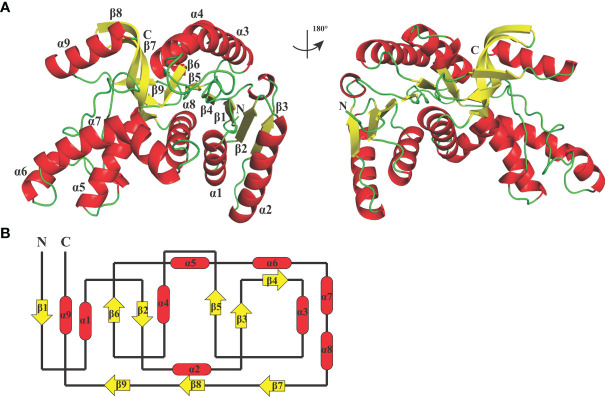
Crystal structure of BoLDH. **(A)** Monomeric structure of BoLDH. The structure of BoLDH was exhibited as a cartoon structure and colored from N-terminal to C-terminal. The α-helicesis and the β-strands were colored in red and yellow, respectively. The mobile β4-α3 loop of BoLDH was shown with black dotted line. **(B)** Topology structure diagram of BoLDH. The topological structure of BoLDH was displayed and the colors were corresponding to that of the panel **(A)** All the cartoons were produced using Pymol viewer 4.30.

**Figure 5:**
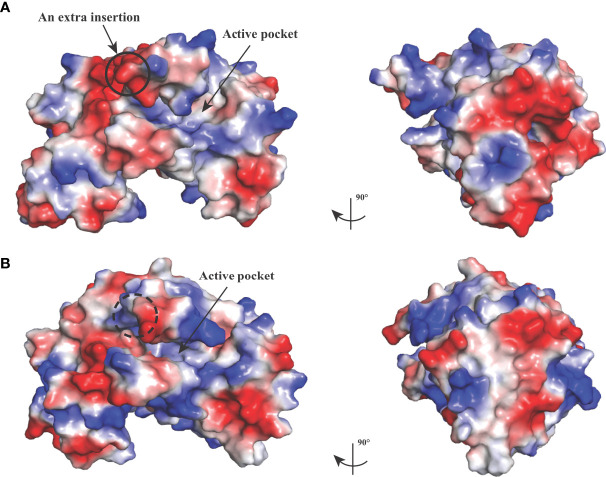
Electrostatic surface potential of BoLDH structure. The crystal structure of BoLDH **(A)** and BmLDH **(B)** in identical orientations are displayed as a molecular surface model colored according to electrostatic potential, and the red regions for negative charge and the blue regions for positive charge are labeled, respectively. Black solid circle indicated the extra insertion of BoLDH, and black dashed circle displayed the corresponding position in BmLDH. The maximum and minimum eV values for the electrostatic map are 64.635 and -64.635, respectively.

The crystal structure of BoLDH apo form (residues Met1 to Gly330) was determined by molecular replacement method and refined to *R*_work_ and *R*_free_ factors of 0.236/0.268 using reflections to 2.67 Å resolution (resolution range, 44.11–2.67 Å). The BoLDH crystals belong to the space group *P*1 with the unit cell parameters *a* = 81.9, *b* = 92.3, and *c* = 109.9 (Å). Two tetramers were observed per asymmetric unit, and each monomer contains a co-factor binding site for the binding of NADH or NAD^+^ and a substrate binding site for the binding of pyruvate or lactate. The details of phasing and refinement are summarized and shown in **Table 1**. As the active-site loop (residues 95–109) in open and disordered state, a poor electron density map for the loop was displayed (**Supplementary Figure S2**). In addition, an extra insertion (position 101–105, —DEEWS—) is present in the active-site loop (β4–α3 loop) of BoLDH and creates a larger catalytic pocket than that of mammalian LDHs (**Figures 1**, **4A**). Interestingly, the absence of this five-peptide insertion makes the BoLDH lose its catalytic function (**Figure 3H**). The secondary structure of BoLDH consists of nine α-helices (α1 to α9), nine β-strands (β1 to β9), and several loops (**Figures 4A, B**). The electrostatic surface potential of BoLDH is visualized in **Figure 5**. Numerous electrostatically neutral and positively charged areas were discovered to be present in the BoLDH catalytic cavity, and the extra insertion produces a red region for negative charge.

### Quaternary Stabilization by the Additional Salt Bridges and Hydrogen Bonds

**Figure 6:**
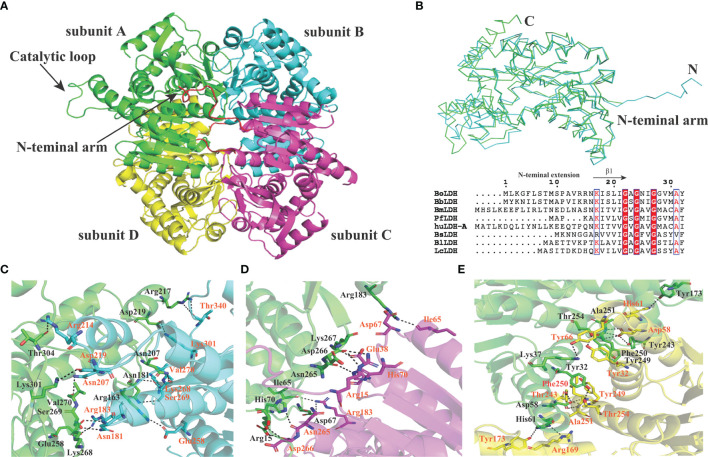
Subunit interfaces of BoLDH. **(A)** The tetrameric state of BoLDH. BoLDH consists of four identical subunits, and colored in green, scan, magenta and yellow, respectively. **(B)** Comparison between apo *Bifidobacterium longum* (Bl) LDH (1lld) and BoLDH. The apo BoLDH (scan) were superimposed on and BlLDH without ligands (green). **(C)** The interface of subunit A and subunit B. Carton models of subunits A and B are displayed in different colors (green and scan). **(D)** The interface of subunit A and subunit C. Carton models of subunits A and C are displayed in different colors (green and magenta). **(E)** The interface of subunit A and subunit D. Carton models of subunits A and D are displayed in different colors (green and yellow). The dashed lines represent the hydrogen bonds and salt-bridges between the respective donor and acceptor atoms. These pictures were produced using the software PyMOL 4.30.

BoLDH is a tetrameric enzyme made of four identical subunits and displays 222 (D2) symmetry (**Figure 6A**). An extension of ~15 residues at the N-terminal, named as N-terminal “arm”, was detected in the apo BoLDH structure, and each “arm” was involved in the formation of an extra salt bridge in the way that a proton transfers from the carboxyl group of residue Asp266 to the guanidine group of residue Arg15 at the interface of subunit A and subunit C or subunit B and subunit D (**Figures 6B, D**). Compared with bacteria allosteric LDHs (such as PDB accession no. 1lld), we found that, without ligands, BoLDH takes R-state with the active-site loop open, and the R-state conformation was more stabilized than that of bacteria allosteric LDHs as the additional salt bridges and hydrogen bonds (**Figures 6C**–**E**). In allosteric LDHs, the absence of the N-terminal “arm” will cause a great decrease on the contact surface of subunits (Abad-Zapatero et al., 1987; Uchikoba et al., 2002). In the study, we artificially removed the N-terminal “arm” (residues 1–15) from BoLDH, but the enzyme missing the N-terminal “arm” retained its catalytic activity (**Figure 3H**). The PfLDH is also considered as a non-allosteric LDH, but it lacks an N-terminal “arm” (**Figure 6B**). The results together suggest that the N-terminal “arm” structure is contributed to the quaternary stabilization of BoLDH but not an essential factor for the stabilization of R-state conformation.

### Structure Alignment of BoLDH, BmLDH, PfLDH, and HuLDH-A

**Figure 7:**
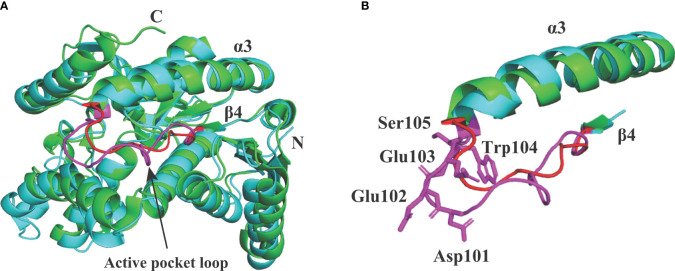
Structural alignment between BoLDH (cyan) and BmLDH (green). **(A)** The apo structure of BoLDH was superimposed on the apo structure of BmLDH (PDB accession number 6k12). The active pocket loop of BoLDH (β4-α3 loop) was shown in magenta and the corresponding region in BmLDH was shown in red. **(B)** Differentiation on the β4-α3 loops from BoLDH and BmLDH. An extra insertion was observed in the active pocket loop of BoLDH but not present in BmLDH. The two loops were colored in magenta and red, and the five amino acid residues in β4-α3 loop of BoLDH were shown in sticks and labeled.

**Figure 8:**
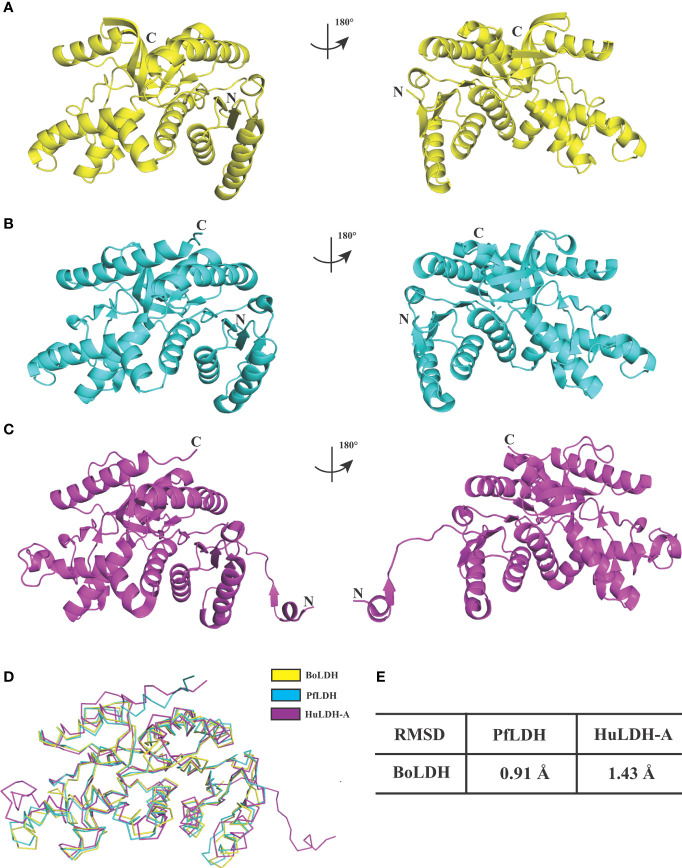
Structural comparisons of BoLDH, PfLDH and HuLDH-A. **(A)** Overlay of BoLDH structure at 180 degrees. **(B)** Overlay of PfLDH structure at 180 degrees (PDB accession number 2x8l). **(C)** Overlay of HuLDH-A structure at 180 degrees (PDB accession number 4ojn). Structures of the three LDHs were exhibited in an identical orientation as cartoons, and colored in yellow, cyan and purple. **(D)** Ribbon diagrams of the three LDH structures. **(E)** Structure identities and RMSD values between BoLDH and PfLDH or between BoLDH and HuLDH-A were shown in the form. The RMSD values were calculated using the PDBeFold service.

In *Babesia* spp. LDHs, the mammalian-like BmLDH contrasts with the typical protozoan-like BoLDH (**Figure 1**). Compared to the BmLDH apo structure (PDB accession no. 6k12), we observed that the overall structure of BoLDH and BmLDH is highly conserved, but their amino acid sequence is a low identity of 27.06%. The five-peptide insertion (residues 101–105) presents in the active pocket loop of BoLDH but not in the BmLDH apo structure, and the specific insertion makes the BoLDH catalytic pocket a little larger than BmLDH (**Figures 7A, B**). For further elaboration on the structural characteristics of BoLDH, we assessed the level of similarity among the secondary structures of those LDH proteins form *B. orientalis*, *P. falciparum*, and *H. sapiens*. Comparing the BoLDH crystal structure with the X-ray crystal structures of PfLDH (PDB accession no. 2x8l) and HuLDH-A (PDB accession no. 4ojn), we observed that the overall structures of the three different LDHs exhibited a much the same fold pattern, including their active pockets and activity-site loops (**Figures 8A**–**C**). The RMSD value between BoLDH and PfLDH (0.91 Å) was lower than that between BoLDH and HuLDH-A (1.43 Å) (**Figures 8D, E**).

### Molecular Model of Compound Gossypol With BoLDH

**Figure 9:**
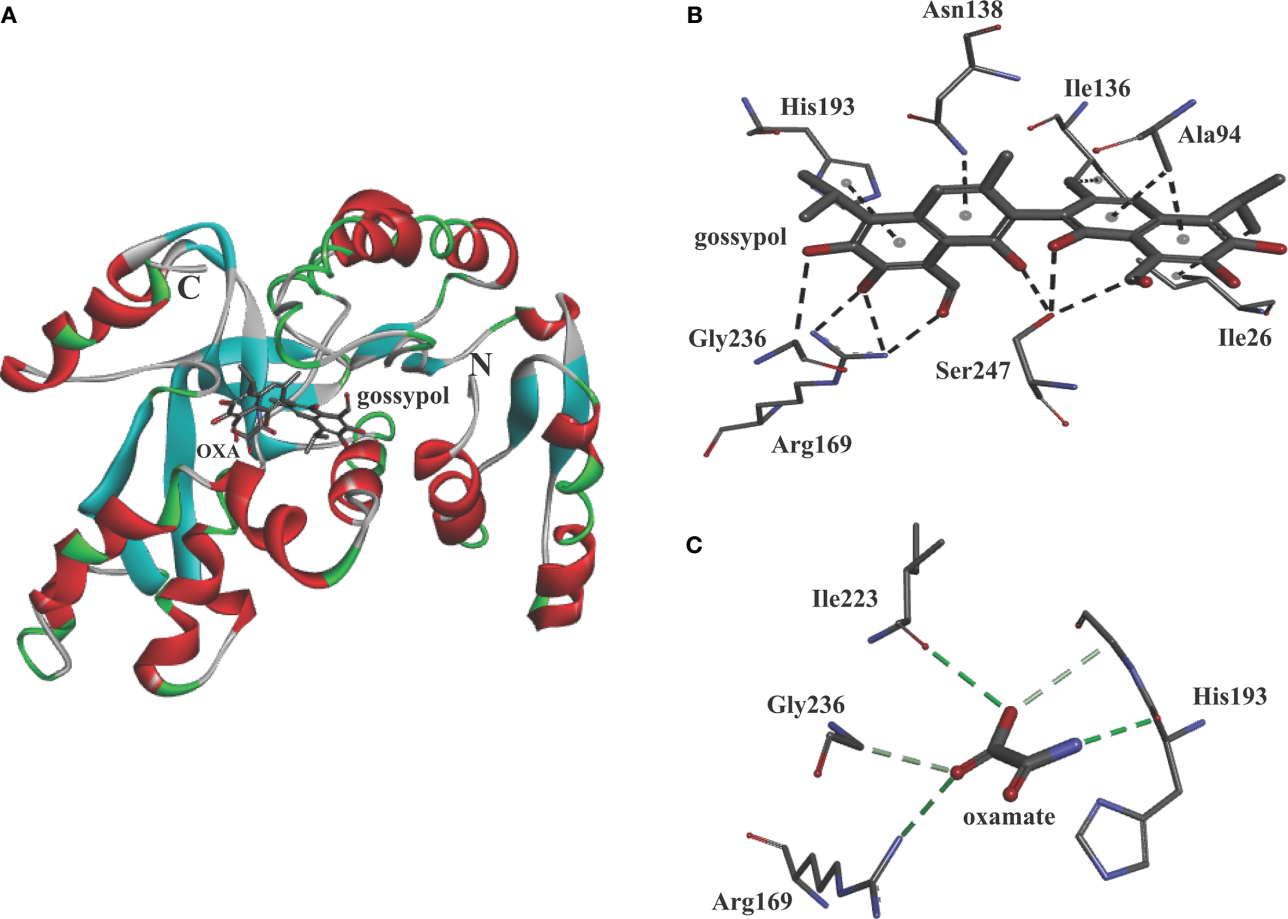
Molecular model of gossypol with BoLDH. **(A)** Structural cartoon of BoLDH with gossypol on NADH binding pocket and oxamate on substrate binding pocket. gossypol and oxamate were displayed as stick diagram. **(B)** The distribution of hydrogen bonds and hydrophobic interactions at the gossypol interface. **(C)** The distribution of hydrogen bonds and hydrophobic interactions at the oxamate interface. The interacting amino acids in the binding network are labeled and shown as stick diagrams, and the dashed lines represent the hydrogen bonds and hydrophobic interaction between the respective donor and acceptor atoms. The two pictures were produced using Discovery Studio 2018 Client software.

For simulating the binding, the compound gossypol was docked into BoLDH crystal structure by using the Discovery Studio software. The docking analysis provided one binding mode for gossypol, and eight amino acid residues (Ile26, Ala94, Ile136, Asn138, His193, Gly236, Arg169, and Ser247) in the active pocket of BoLDH were involved in the binding of gossypol (**Figures 9A, B**). However, the key for the strong binding (inhibition) of gossypol is still not clear. We speculate that it may be driven by the formation of Schiff’s bases *via* the interaction between the aldehydic groups of gossypol and lysine (K) or arginine (R) residues of the proteins or in the way of the hydrogen bond formation with the poly-phenolic hydroxyl groups. In the binding mode for gossypol, a conventional hydrogen bond was observed between the guanidinium group of residues Arg169 and the carbonyl group of gossypol. Hence, the formation of Schiff’s bases could be a convictive explanation for the strong binding of gossypol. The reported co-crystal structures (PfLDH: 1t2d and BmLDH: 6k13) display that several highly conserved amino acid residues participate in LDH-oxamate binding (**Figure 1**). Hence, the substrate analog oxamate was used as a control in the docking analysis, and we found that four amino acid residues (Arg169, His193, Ile223, and Gly236) were involved in the binding of BoLDH and oxamate (**Figure 9C**). The LDH-oxamate binding model further supports the model of gossypol with BoLDH as the residues Arg169 and His193 are identical across all apicomplexan LDHs.

## Discussion

In the present study, we identified a novel anaerobic metabolic enzyme with ~40-kDa size, and the enzyme was named as BoLDH because the amino acid sequence of BoLDH exhibited an identity of ~90% with BbLDH and several conserved LDH motifs. The crystal structure of BoLDH further revealed that the enzyme had a high degree of structural conservation with other protozoan LDHs, including PfLDH and TgLDH, especially a representative penta-peptide insertion found in the opened and disordered loop of other protozoans and which was also detected as present in BoLDH. These results together suggested the identification of the new enzyme in *B. orientalis* glucose metabolism as precisely *B. orientalis* LDH.

Interestingly, we found that the structure of apo BoLDH takes R state with the active-site loop (β4–α3 loop) open, and the feature is similar to other eukaryotic apo LDHs, such as PfLDH, HuLDH-A, and BmLDH, but obviously different from that of bacterial LDHs in R state with the active-site loop closed (**Supplementary Figure S3**). Hence, BoLDH belongs to a group of non-allosteric L-LDHs. In the study, we also attempted to determine the crystal structure of the BoLDH complex with NADH and oxamate (pyruvate analogue). Although we successfully acquired the crystal complex with NADH and oxamate, the co-crystal showed a very bad crystal quality. However, in *P. falciparum*, the crystal structure of the PfLDH complex with NADH and oxalate has been solved at 1.1-Å resolution (PDB accession no. 1t2d). Nine residues (Met25, Ile26, Asp46, Try78, Gly92, Phe93, Val136, Asn138, and Leu161) in the PfLDH activity cavity were observed to participate in co-factor NADH binding. By comparing the amino acid sequence of PfLDH with other protozoan LDHs, five residues (Ile26, Asp46, Try78, Gly92, and Asn138) displayed a complete identity across all apicomplexan LDHs (**Figure 1**). Therefore, we speculate that the five residues could play a key role in the catalytic reaction of BoLDH. This speculation needs to be validated by further studies.

In the LDH family, the dynamic β4–α3 loop gates the catalytic pocket of LDHs and plays a crucial role in their catalytic process (Nie et al., 2016). Interestingly, the protozoal LDHs have a five-peptide insertion in the catalytic pocket loop, except for *B. microit* LDH, and the extra insertion was not involved in the binding of co-factor/substrates in PfLDH complex structure (PDB accession no. 1t2d). Therefore, the specific insertion could help these protozoal LDHs to better adapt to other substrates and cofactors, such as the substrates 2-ketobutyrate and hydroxypyruvate and the co-factor APADH. For confirming the hypothesis, we remove the extra five-amino-acid residues from the catalytic loop of BoLDH to create a similar loop size to that of mammalian LDHs. However, the deletion of this five-peptide insertion results in the loss of function of BoLDH. As the catalytic loop does not participate in the subunit interactions of BoLDH, therefore, we infer that the extra insertion could affect the closure of the catalytic pocket. The results together suggest that the development of compounds for aborting the movement of these dynamic loops could be a novel strategy for designing LDH inhibitors.

Compared to the kinetic parameters of PfLDH, rBoLDH has the ability to catalyze the reversible conversion of pyruvate to lactate by using β-NADH or NAD^+^, but the catalytic rates are lower than PfLDH (Gomez et al., 1997). The *K*_M_ values of rBoLDH for pyruvate, NADH, and NAD^+^ are 102, 126, and 493 μM, and these values are higher than those of the values of PfLDH (30 μM for pyruvate, 7 μM for NADH, and 86 μM for NAD^+^). However, the *K*_M_ value of rBoLDH for lactate (166 μM) was ~72-fold lower than that of PfLDH (12,000 μM for lactate). In the study, the NAD^+^ analog APAD^+^ was used by BoLDH to catalyze the lactate to pyruvate with ~3-fold higher *K*_cat_ value than NAD^+^, and using APAD^+^ as cofactor, BoLDH exhibited ~13-fold lower *K*_M_ for lactate (**Table 2**). Interestingly, in the erythrocytic LDH isoenzyme, it is very hard to use the 3-acetyl pyridine analog (Makler and Hinrichs, 1993). Therefore, *Babesia* spp. LDHs could serve as a potential antigen molecule for the development of a simple diagnostic assay for the detection of *Babesia*.

Gossypol, with a structure of six phenolic hydroxyl and two aldehydic groups, has a powerful inhibitory effect on various oxidoreductases (Gerez de Burgos et al., 1984; Keshmiri-Neghab and Goliaei, 2014). However, at high concentrations, gossypol revealed a major toxicity against the mammalian cell, especially spermatocytes (HCT-8 cells, IC_50_ = 51 μM) (White et al., 1988; Bork et al., 2004; Zhang et al., 2015). As a result, it is significant to screen and design highly selective compounds (the core group of gossypol) based on the structure of *Babesia* spp. LDH, but no structural data is provided. In this paper, we first provide a crystal structure of BoLDH at 2.67-Å resolution, and the structural model would offer some structural basis for the development of new LDH inhibitors. With the development of computer technology, drug discovery has ushered in the era of virtual screening, and potential drugs are predicted by docking and molecular dynamics simulations on the target and drug candidates to calculate the affinity between ligand and potential drugs. In malaria parasites, the method of virtual screening has been used extensively for finding and designing new therapeutic drugs (Brandao et al., 2018; Saxena et al., 2019). However, the method has not been applied for the development of anti-babesial drugs. In the following work, a virtual drug screening based on the BoLDH crystal structure and a known drug library to find new BoLDH inhibitors could be a future scope.

Overall, we successfully isolated a novel cDNA clone for encoding BoLDH, elaborated its biological characteristics, and reported the crystal structure of apo BoLDH. We further show that the phenolic aldehyde gossypol inhibits BoLDH activity and suggest the renewed structure for gossypol to develop derivatives of this compound. Our studies will set the stage for future efforts to design LDH-specific inhibitors that exploit the uniqueness of LDH enzymes and the vulnerabilities of *Babesia* spp. for the development of new classes of anti-babesiosis drugs.

## Data Availability Statement

The nucleotide sequence and the structure data generated during this study were separately submitted to the NCBI GenBank and the Protein Data Bank (PDB) under the accession number MW412839 and 7W8A.

## Author Contributions

LY, HA, LH, and JZ designed the study and wrote the draft of the manuscript. QL and WL performed the experiments and analyzed the results. All authors contributed to the article and approved the submitted version.

## Funding

This work was supported by the National Natural Science Foundation of China (Grant No. 31930108 and 31772729), China Postdoctoral Science Foundation funded project (Grant No. 2020M682448), the Fundamental Research Funds for the Central Universities, China (Grant No. 2662020DKPY016 and 2662019PY001).

## Conflict of Interest

The authors declare that the research was conducted in the absence of any commercial or financial relationships that could be construed as a potential conflict of interest.

## Publisher’s Note

All claims expressed in this article are solely those of the authors and do not necessarily represent those of their affiliated organizations, or those of the publisher, the editors and the reviewers. Any product that may be evaluated in this article, or claim that may be made by its manufacturer, is not guaranteed or endorsed by the publisher.

## Abbreviations

LDH, lactate dehydrogenase; MDH, malate dehydrogenase; TCA cycle, Krebs cycle; RMSD, root mean square deviation; ATP, adenosine triphosphate; PDB, Protein Data Bank; NAD^+^, nicotinamide adenine dinucleotide; APAD^+^, 3-acetylpyridine-adenine dinucleotide; β-NADH, β-nicotinamide adenine dinucleotide, reduced; APADH, 3-acetylpyridine-adenine dinucleotide, reduced.
