# Supplementary material for: The Structural Basis of Babesia orientalis Lactate Dehydrogenase
Source: Front Cell Infect Microbiol. 2022 Jan 5;11:790101. doi: 10.3389/fcimb.2021.790101 (PMC8766848; doi:10.3389/fcimb.2021.790101)
Supplement: Supplementary file 1 [file DataSheet_1.docx]

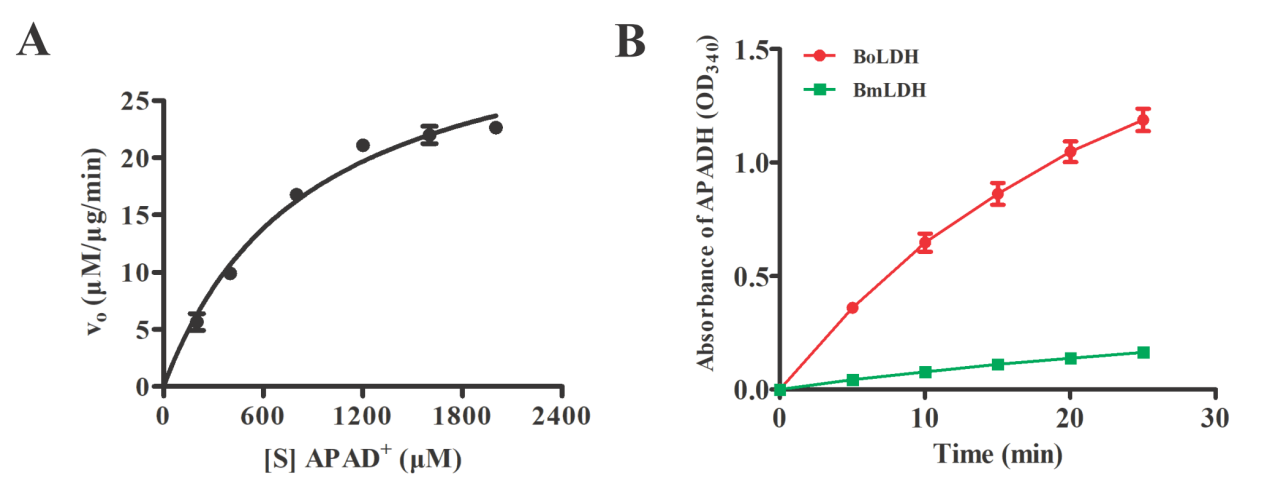


**Supplementary Figure S1: (A)** Michaelis-Menten Kinetics for the reverse reaction of rBmLDH. Variations of rBoLDH activity with concentration of APAD^+^. **(B)** Enzyme catalytic curves based on APAD^+^ as a co-factor. The concentrations of substrate and co-factor are at saturation, and the productions of APADH were measured at OD_340._ The error bar represents mean ± SD (n=3), and Non-linear regressions and XY graph were drawn with GraphPad5.0.


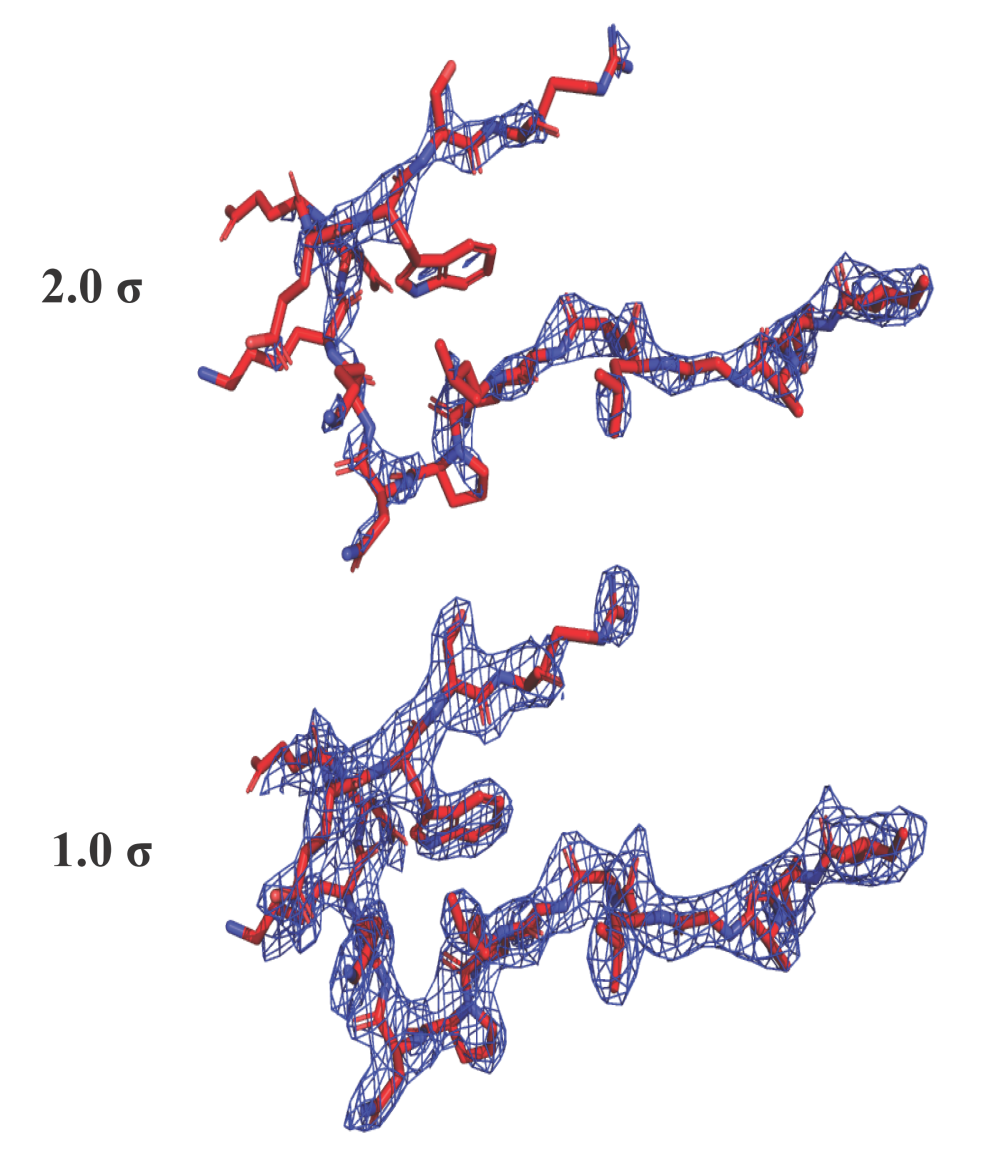


**Supplementary Figure S2.** 2*Fo-Fc* electron density maps for the activity pocket loop in apo BoLDH at 2.0 σ and 1.0 σ. A poor density map was displayed in apo BoLDH structure because of the swing.

**
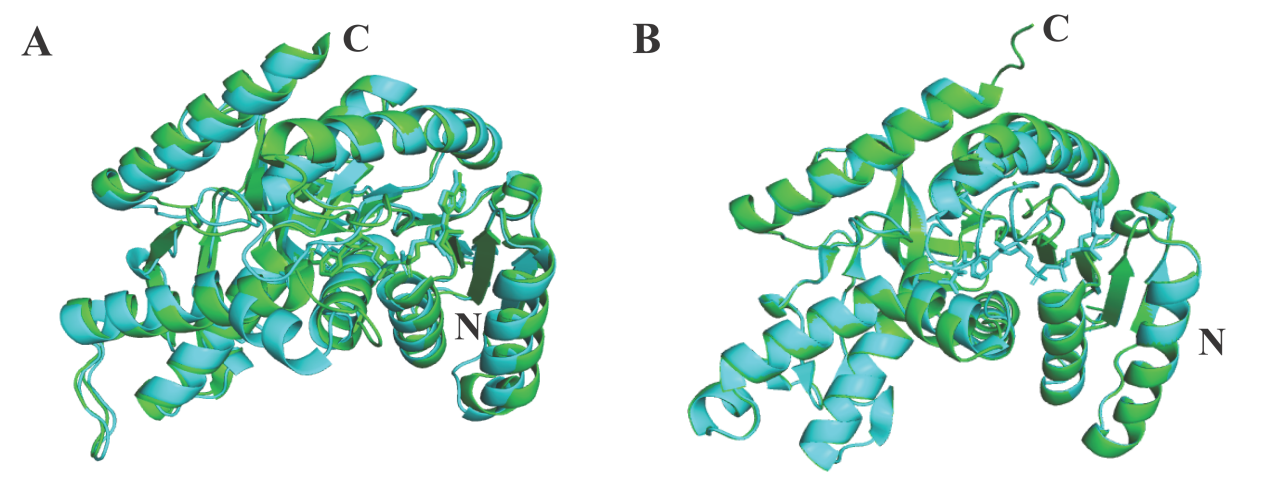
**

**Supplementary Figure S3.** (A) Comparison between two state structures of bacteria LDHs. The R state of *Bifidobacterium longum* LDH with NADH, oxamate and fructose 1,6-bisphosphate (FBP) (1LTH, cyan) were superimposed on T state of *Bifidobacterium longum* LDH with NADH (1LLD, green). (B) Comparison between apo PfLDH (2x81) and holo PfLDH (1t2d). PfLDH with ligands (cyan) were superimposed on and PfLDH without ligands (green).

**TABLE S1 |** Oligonucleotide primers used for amplifying the full ORF of BoLDH.

| **Primers** | **Primer sequences (5’-3’)** | **Restriction enzyme** |
| --- | --- | --- |
| BoLDH-F | GCGGATCCATGCTCAAAGGTTTTTTATCC | *BamH* I |
| BoLDH-R | AGCTCGAGTTAACCAGCAAGGGCCTCCAATC | *Xho* I |
